# Epiretinal Amniotic Membrane Influences the Cellular Behavior of Profibrotic Dedifferentiated Cells of Proliferative Vitreoretinopathy *In Vitro*

**DOI:** 10.1155/2023/8820844

**Published:** 2023-10-18

**Authors:** Anna Hillenmayer, Laura D. Strehle, Christina Hilterhaus, Andreas Ohlmann, Christian M. Wertheimer, Armin Wolf

**Affiliations:** ^1^Department of Ophthalmology, University of Ulm, Prittwitzstr. 43, Ulm 89075, Germany; ^2^Department of Ophthalmology, Ludwig-Maximilians University, Mathildenstr. 8, Munich 80336, Germany

## Abstract

Proliferative vitreoretinopathy (PVR) as a rare fibrotic ocular disease is the main reason for failure of retinal detachment surgery and a reduced prognosis following surgery. Amniotic membrane (AM) is a versatile surgical tool for tissue stabilization, antifibrotic properties, and regeneration. Initial clinical case studies now demonstrated intravitreal tolerance as well as good anatomical and functional results for degenerative retinal diseases. Due to its diverse wound healing properties, AM could have promoting, suppressive, or no effects on PVR. To illuminate the potential of epiretinal AM transplantation in complex retinal detachment cases, we investigated its influence on human primary PVR (hPVR) cells *in vitro*. In our cell culture study, hPVR cells were isolated from surgically removed PVR membranes. Following incubation with AM for 48 h, AM-incubated hPVR showed significantly reduced proliferation (BrdU-ELISA; *p* < 0.001), migration (Boyden chamber, scratch assay; *p* = 0.003 and *p* < 0.001), and cell adhesion (*p* = 0.005). Collagen contraction was nearly unaffected (*p* = 0.04), and toxicity (histone-complexed DNA ELISA, WST-1 assay, and life/dead staining) was excluded. Next, immunofluorescence showed a myofibroblastic phenotype with reduced expression of fibrosis markers in AM-incubated cells, which was confirmed by Western blot analysis. In the proteomics assay, AM significantly regulated proteins by a more than 2-fold increase in expression which were related to the cytoskeleton, lipid metabolism, cell-matrix contraction, and protein folding. In conclusion, this *in vitro* work suggests no induction of fibrosis and other key steps in the pathogenesis of PVR through AM but rather inhibiting properties of profibrotic cell behavior, making it a possible candidate for suppression of PVR. Further clinical studies are necessary to evaluate the therapeutic relevance.

## 1. Introduction

Proliferative vitreoretinopathy (PVR) is a severe complication following rhegmatogenous retinal detachment and is associated with a poor visual prognosis [1]. Currently, the only available therapeutic option is pars plana vitrectomy, an invasive microsurgical procedure that allows access to the posterior segment of the eye and the vitreoretinal interface through small incisions in the pars plana region of the eye [2]. However, it is common for surgery to fail to provide lasting improvement and repeated procedures are often required. Therefore, in addition to surgery, a treatment method that inhibits the pathologic formation of PVR would be desirable [3].

Many different ocular tissues and cells are involved, such as retinal pigment epithelial (RPE) cells, Müller cells, and immune cells, which physiologically have critical functions in maintaining retinal homeostasis and immune responses. PVR is the result of retinal tears and detachments and a breakdown of the blood-retinal barrier, retinal hypoxia, and increased intraocular inflammation with release of cytokines, growth factors, and chemokines [3]. The cells then undergo mesenchymal transformation to myofibroblasts and form tractional membranes that cause recurrent retinal detachment [4].

Amniotic membrane (AM) transplantation is an established and popular treatment option for ocular surface reconstruction. In addition to its good biocompatibility, it has antineovascular, anti-inflammatory, antiapoptotic, and immunomodulatory effects that inhibit scar formation and prevent pathologic wound healing [5] and thus promote the restoration, regeneration, and stabilization of a physiologic ocular surface [6]. These properties make it attractive for intraocular implantation, and its use has been positively demonstrated in several clinical case series for various atrophic vitreoretinal diseases [7–9]. These studies showed promising regenerative effects and stabilization of degenerative retinal tissue [10].

However, compared to atrophic retinal diseases, PVR is caused by a severe fibrotic cell response to retinal detachment [11, 12]. Despite the different pathogenesis, first intraocular implantations of AM in case series with complex retinal detachments at high risk for PVR development also indicated a beneficial effect [13, 14]. Other studies showed that AM may promote wound healing through fibroblast proliferation and induction of migration and expression of the extracellular matrix [15]. Thus, the exact effects of epiretinal AM application on PVR remain unclear at the cellular and molecular level. Hypothetically, this effect is concentration and interaction dependent; these growth factors may have a dedifferentiating and proliferative effect on different cell types [5, 16, 17], and it is possible that they may also promote a fibrotic response depending on the dominant cell type and surrounding tissue environment [18, 19].

As an *in vitro* study on this topic, we aim to determine if AM has the potential to inhibit and attenuate the pathological progression of primary isolated human PVR (hPVR) cells derived from surgically removed PVR membranes in terms of their profibrotic cell behavior, phenotypic changes, and protein expression pattern by immunofluorescence staining, protein expression by Western blotting and proteomics analysis, cell migration, and proliferation analysis. Ultimately, a better pathophysiological understanding of *in vitro* could lead to a potential clinical application.

## 2. Methods

### 2.1. Surgical Procedure

All cell culture experiments were performed with primary human proliferative vitreoretinopathy (hPVR) cells. Cell isolation was performed as previously described [20, 21]. Pars plana vitrectomy for rhegmatogenous retinal detachment with concomitant PVR was performed in 4 patients. Membranes were carefully removed using 0.25 mg/ml brilliant blue vital dye (Fluoron GmbH, Neu-Ulm, Germany) with terminal forceps (D.O.R.C, Zuidland, The Netherlands). Removal of the tractive PVR membranes did not affect the surgical technique, as they must always be removed for the above diagnosis. All patients gave their informed written consent for PVR membrane collection and research. Furthermore, the study was approved by the ethics committees of the Universities of Ulm and Munich (Ethics Committee of the University of Ulm, approval ID: 26420, and Ludwig-Maximilians-University of Munich, approval ID: 47114), and guidelines of the Declaration of Helsinki were followed.

### 2.2. Cell Culture

No later than three hours after surgical removal, PVR membranes were attached to cell culture plastic (Sarstedt, Nürmbrecht, Germany) with entomological pins (Entomoravia, Slavkov u Brna Czech Republic) under slight tension and suspended in Minimum Essential Medium (MEM, Gibco life technologies, Carlsbad, CA, USA) containing 10% fetal calf serum (Sigma Aldrich, St. Louis, USA). After the hPVR cells had grown from the membrane onto the cell culture plastic, the concentration of fetal calf serum was reduced to 2%, where it remained for all following experiments. The medium was replaced every other day.

### 2.3. Experimental Setup

For all experiments, cell culture inserts (Corning, Arizona, USA) with a porous membrane (0.4 *µ*m) were suspended over the cells for 48 hours. They either contained cryopreserved fresh AM not suitable for transplantation purposes (Tissue bank of the University of Ulm, Ulm, Germany) or were empty for the control group. If an experiment had to be performed in a 96-well cell culture plate, the AM was added directly to the medium.

### 2.4. Proliferation

A BrdU cell proliferation ELISA kit (Roche Diagnostics GmbH, Mannheim, Germany) was used. Briefly, cells were incubated in their experimental groups as described above, followed by another 48-hour incubation with medium containing BrdU labeling solution. All further steps followed the manufacturer's protocol exactly. Absorbance was measured by the photometer (Tecan infinite M200 pro, Tecan, Männedorf, Switzerland) at a wavelength of 450 nm and a reference of 690 nm. The test was performed three times, with each reading recorded twelve times per group.

### 2.5. Scratch Assay

hPVR cells were cultured to complete confluence and scratched with a 100 µl pipette tip (Brand, Lippstadt, Germany). Subsequently, cell-free areas were documented at 0 and 24 hours using an inverted phase-contrast microscope (Zeiss Axiovert 35, Jena, Germany) and a digital camera (Nikon D31000, Tokio, Japan). ImageJ 1.53 k (National Institutes of Health, Bethesda, Maryland, USA) was used to evaluate the repopulated area as a percentage of the cell-free area at 0 hours.

### 2.6. Boyden Chamber

Cell migration was observed using a Boyden chamber setup. The upper chamber was a cell culture insert with a membrane (Corning) with pores of 8 *µ*m in diameter. The insert was suspended into the well of a cell culture plate which acted as the lower chamber. 1.1 × 10^5^ cells/cm^2^ were filled into the top chamber, and medium was added to the setup. Chemotaxis was induced by placing AM in the lower chamber. Cells were able to migrate for 5 hours under standard cell culture conditions with and without AM. After fixation with methanol at 4°C for 20 min, a Giemsa Azur-Eosin-Methylene Blue solution (Merck Millipore) was used to make cells visible. Subsequently, all nonmigrated cells from inside the cell culture insert were removed by a cotton bud. Documentation was followed under an inverted phase-contrast microscope with a camera (Zeiss Axiovert 35 and Nikon D31000). Migrated hPVR cells were counted manually using ImageJ 1.53 k (NIH, Bethesda, MD, USA).

### 2.7. Extracellular Matrix Contraction

A collagen-based cell contraction assay (Cell Biolabs, San Diego, CA, USA) was used according to the manufacturer's instructions. In brief, 500 *µ*l of gel containing 2 × 10^6^ hPVR cells was polymerized in a 24-well plate.

After 1 hour, medium was added and the collagen gels were exposed to AM or the control setting. After 2 days, the collagen gels were detached from the edge of the cell culture plate using a spatula. Photo documentation was performed daily starting after the detachment of the gel, and spatial dimensions were determined on the images measuring the surface area using ImageJ 1.53 k (NIH).

### 2.8. Adhesion

Cells were treated as described above and then subcultured. Successively, a cell concentration of 1 × 10^3^ cells/cm^2^ was resuspended in medium and photographed 30, 60, and 120 minutes later under the inverted phase-contrast microscope. Already attached cells were counted manually on the images at the different time points.

### 2.9. Immunofluorescence Staining

For immunofluorescence characterization, freshly extracted PVR membranes were fixed in 4% formaldehyde solution (Invitrogen, Waltham, MA, USA) overnight. Following three washes with 0.1 M phosphate-buffered saline (PBS, Thermo Fisher Scientific, Waltham, MA, USA), the membranes were fixed with entomological pins to a cell culture dish and incubated with blocking solution (3% bovine serum albumin, 0.1% triton X 100 in 0.1 M phosphate buffer) for blocking of unspecific antigen staining. All further steps followed the same protocol as the one used for cell staining.

For cell staining, sterile microscopy coverslips (Menzel-Gläser, Braunschweig, Germany) were placed on the cell culture plastic and hPVR cells were cultured on top. All further experimental procedures were then performed on the coverslips. After incubation with AM, cells were washed three times with PBS and fixed with 4% formaldehyde solution. Three washing steps were performed between all experimental steps. Permeabilization was performed by adding 0.25% Triton X-100 (Roche) in PBS for 10 minutes. Then, 1 : 10 Roti Immunoblock (Carl Roth, Karlsruhe, Germany) in distilled water was added to prevent nonspecific antibody binding. Diluted in 1 : 10 blocking solution in PBS, the incubation with the primary antibodies is followed: cytokeratin-8 rabbit antihuman (1 : 250, Abcam AB53280, Cambridge, United Kingdom), fibronectin rabbit antihuman (1 : 50, Abcam AB268020), vimentin rabbit antihuman (1 : 200, Abcam AB92547), glial fibrillary acidic protein rabbit antihuman (1 : 500, DAKO Z0334, Agilent, Santa Clara, CA, USA), Iba1 rabbit antihuman (1 : 750, Wako 019–19741, Richmond, VA, USA), CD45 mouse antihuman (1 : 250, Santa Cruz SC-20056, Dallas, Texas, US), and CD68 mouse antihuman (1 : 50, Abcam AB955). An alexa fluor 594 conjugated antibody was used for CRALBP mouse antihuman (1 : 200, Santa Cruz SC-59487) and a Cy3-conjugated *α*-SMA mouse antihuman (1 : 300, Sigma Aldrich C6198, St. Louis, MS, USA). Incubation took place overnight at 4°C and an hour incubation with the secondary antibody ensued with alexa fluor 595 coupled goat antimouse (1 : 500, Thermo Fisher Scientific) or alexa fluor 595 or alexa fluor 488 coupled goat anti rabbit (1 : 500, Thermo Fisher Scientific). Alexa Fluor 488 coupled phalloidin (1 : 400, Invitrogen A12379) was incubated at room temperature for one hour without the need of a secondary antibody incubation. Finally, a drop of ProLong Gold antifade reagent containing DAPI (Invitrogen) was dropped onto a slide (Epredia, Braunschweig, Germany). The PVR membranes were then whole mounted onto the glass slides. Cover slips were placed on top of the membranes or directly containing cells and allowed to set at 4°C for 24 hours. Staining was recorded using a fluorescence microscope with camera (DM4000B, Leica, Wetzlar, Germany).

### 2.10. Liquid Chromatography with Tandem Mass Spectrometry-Based Proteomic Analysis

A proteome analysis was performed as described previously [22] in collaboration with the Core Unit Mass Spectrometry and Proteomics of the University of Ulm under the direction of Dr. Sebastian Wiese. Samples were prepared as described above, frozen at −80 degrees, and then transferred to liquid nitrogen. After the transport to the Core Unit, cells were washed and then lysed in DIGE buffer (30 mM Tris base, 7 M urea, 2 M thiourea, and pH 8.5). For sample purification, all samples were precipitated by methanol/chloroform extraction according to known protocols [23]. 4 *µ*g of each sample was reduced with 5 mM DTT (AppliChem, Darmstadt, Germany) for 20 min at RT, alkylated with iodoacetamide (Merck Millipore) for 20 min at 37°C, and diluted with 50 mM ammonium bicarbonate. Trypsin was added at a 1 : 50 enzyme to protein ratio and digested overnight at 37°C. Samples were measured using an LTQ Orbitrap Elite system (Thermo Fisher Scientific) coupled online to a U3000 RSLCnano (Thermo Fisher Scientific) as previously described [24] with the following modifications: the column was first equilibrated in 5% B for 5 min (solvent: A 0.1% FA; B 86% ACN, 0.1% FA). This was followed by various elution steps in which the amount of B was first increased from 5% to 15% in 5 min and then from 15% to 40% B in 145 min. The 20 most intense ions from the survey scan were selected for CID fragmentation. Singly charged ions were discarded, and the m/z of fragmented ions was excluded from fragmentation for 60 seconds. MS2 spectra were acquired with the LIT at fast scan speeds. Database searches were performed using MaxQuant ver. 1.6.3.4 [25]. For peptide identification, the Andromeda integrated search engine was used to correlate MS/MS spectra with the UniProt human reference proteome set. Carbamidomethylated cysteine was considered as a fixed modification along with oxidation (M) and acetylated protein N termini as variable modifications. The false discovery rate was set at 0.01 at both the peptide and protein levels.

### 2.11. Western Blot

A Western blot was performed as described previously [26]. In brief, whole cell extracts were prepared from the pretreated hPVR. For this purpose, the medium and AM were removed, and cells were washed, scrapped of the cell culture plastic, and afterwards centrifuged twice in cold PBS containing magnesium chloride. The resulting cellular material was suspended in lysis buffer 17 (Bio-Techne, Minneapolis, MN, USA) containing protease inhibitor C 100X Halt cocktail (Thermo Fisher Scientific) and phosphatase inhibitor cocktail (Merck Millipore). The hPVR cells were shaken on ice for 30 minutes before a 30-minute centrifugation. Both the cell extract and the supernatant were frozen at −80°C. Protein concentration detection was performed using a Pierce BCA Protein Assay Kit (Thermo Fisher Scientific) as detailed by the manufacturer. Following the results of the BCA protein detection, the samples were diluted using lysis buffer before performing gel electrophoresis to ensure that all samples finally contained an equilibrated amount of total protein. Lysates were diluted in 4x Laemmli Sample Buffer (Bio-Rad, Hercules, CA, USA) containing 100 mM 1,4-dithiothreitol and then separated by electrophoresis in a 4–20% Mini Protean TGX Precast Gel (Bio-Rad) using Tris-Glycin-SDS buffer (Bio-Rad) for 41 min at 200 V and 0.4 A. Afterwards, proteins were transferred by electroblotting to a polyvinylidene difluoride membrane (Bio-Rad) using a PerfectBlue Semi-Dry-Blotter (PEQLAB, VWR, Darmstadt, Germany). A current of 0.08 A was set for 90 minutes for the transfer, and the voltage was limited to a maximum of 90 volts. Next, membranes were cut into pieces according to the desired molecular weights of the following antibodies. After blocking overnight in 0.05% blocking reagent (Roche) in 0.001% Tween-20 (Bio-Rad) in PBS without Mg^2+^ and Ca^2+^ (Thermo Fisher Scientific), the following primary antibodies were diluted in antibody dilution solution (1 : 4 blocking solution in PBS): alpha-smooth muscle actin rabbit antihuman (1 : 2000, Merck Millipore A5228), cytokeratin-8 rabbit antihuman (1 : 10.000, Abcam AB53280), fibronectin rabbit antihuman (1 : 1000, Abcam AB268020), and vimentin rabbit antihuman (1 : 2500, Abcam AB92547). Subsequently, the membranes were incubated on a shaker with the primary antibody for 75 minutes and 30 minutes with the secondary antibodies that were horseradish peroxidase conjugated: mouse IgG solution (1 : 20.000 Biorad 170–5047) and rabbit IgG (1 : 30.000 Biorad 170–5046). Pierce ECL Plus Western blotting substrate (Thermo Fisher Scientific) was used as detailed by the manufacturer, and chemiluminescence signals were detected by the imaging system Fusion Pulse TS (Vilbert Lourmat, VWR). To strip the former incubated membranes, membranes were washed several times and incubated with Restore Plus Western blotting stripping buffer (Thermo Fisher Scientific) for 45 minutes. After washing the membranes again, incubation with the primary antibodies actin mouse antihuman (1 : 5000, Novus NBP2-25142), HSP-70 mouse antihuman (1 : 1000, abcam ab5439), alpha tubulin IgG mouse antihuman (1 : 10.000 Abcam, ab7291) followed. All following steps are identical to the ones described above.

### 2.12. Histone-Complexed DNA ELISA

For the detection of apoptosis in hPVR, an ELISA to detect histone-DNA complexes (Cell Death Detection ELISA, Roche) was performed after 48 hours of incubation with AM in accordance with the manufacturer's recommendations using a cell density of 1,25 × 10^4^ cells/cm^2^. Absorbance was measured at a wavelength of 405 nm including a reference at 490 nm by the Tecan infinite M200 pro (Tecan).

### 2.13. Life/Dead Staining

hPVR cells were seeded onto coverslips. Slides incubated with methanol for 10 min at 4°C served as a positive control. Treatment was followed by incubation with Hoechst (Invitrogen, Waltham, MA, USA) 1 *μ*g/ml and propidium iodide (Invitrogen) 2 *μ*g/ml in medium for 15 min. After three washing steps, cells were fixed using 4% formaldehyde solution (Invitrogen) for 10 min. Following another three washings steps for 5 minutes each, the slides were mounted on slides using antifade mounting medium (Vectashield, Vector laboratories, Newark, CA, USA). Images were acquired using a fluorescence microscope equipped with a camera (DM4000B, Leica, Wetzlar, Germany).

### 2.14. WST Assay

A colorimetric dye reduction assay was conducted as detailed by the manufacturer (WST-1, Roche Diagnostics, Mannheim, Germany). The same experimental settings were used as described above with a cell density of 2.5 × 10^4^ cells/cm^2^. WST-1 was added to the cell culture medium for 60 minutes, and subsequently, absorbance was measured at a wavelength of 450 nm and a reference of 690 nm.

### 2.15. Statistical Analysis

Data collection and calculations were done in EXCEL 365 (Microsoft, Redmond, Washington). Statistical analysis was performed with SPSS 28 (IBM, Armonk, United States) and GraphPad PRISM 9 (GraphPad Software, Inc. San Diego, California). All quantitative assays consisted of either 3 or 4 biological replicates with at least 3 or more technical replicates each. The results from the technical replicates were used for statistical testing. A two-tailed *t*-test was used for all assays comparing two groups (BRDU cell proliferation, scratch assay, adhesion, Boyden chamber, extracellular matrix contraction, Western blot, and WST). A *t*-test was also performed for proteomics. For more than two groups (histone-complexed DNA ELISA), ANOVA with post hoc LSD test was used. Data are presented as the mean and error bars as the standard deviation. *P* values <0.05 were considered statistically significant.

## 3. Results

### 3.1. Proliferation

**Figure 1:**
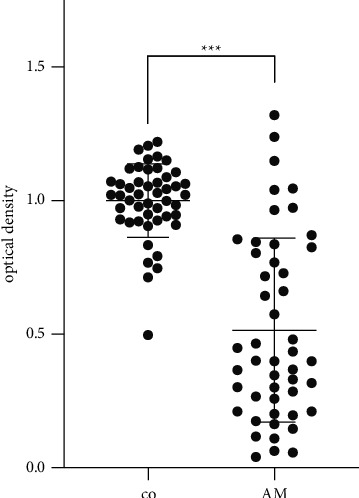
Addition of AM resulted in a statistically significant decrease in cell proliferation compared to the unexposed control in the BrdU assay (^*∗∗∗*^*p* < 0.001; *n* = 48 technical repeats of 4 independent biological experiments).

In PVR formation, stimulation of proliferation of uncontrolled transdifferentiated myofibroblastic cells (hPVR) leads to rapid disease progression. Cell proliferation of hPVR, as measured by the BrdU assay, was statistically significantly reduced (*p* < 0.001; *n* = 48 technical repeats of 4 independent biological experiments) when AM was added to the culture (Figure 1).

### 3.2. Cell Migration

**Figure 2:**
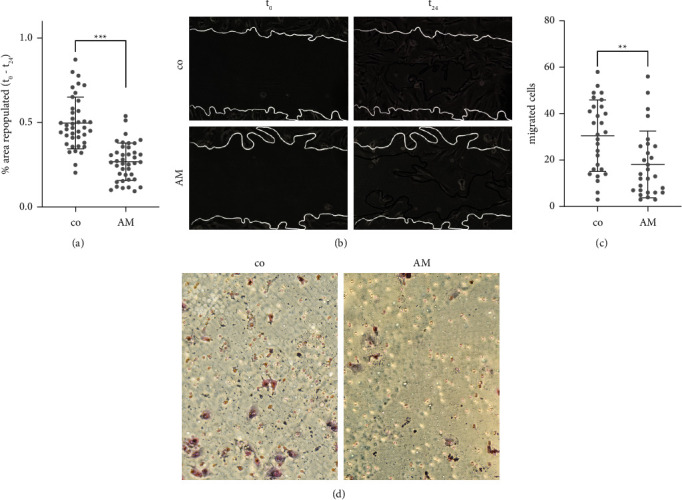
Cell migration was attenuated in both (a) the scratch (*p* < 0.001) and (c) Boyden chamber assays (*p* = 0.003). (b) Photographs of the scratch assay were taken directly after scratching (*t*_0_) and 24 hours later (*t*_24_). White lines represent the margins at 0 hours and black lines at 24 hours. (d) Cells which migrated through the membrane were stained with Giemsa azure-eosin methylene blue solution (^*∗∗*^*p*=0.003; ^*∗∗∗*^*p* < 0.001; scratch assay: *n* = 40 technical repeats of 4 independent biological experiments; Boyden chamber: *n* = 28 technical repeats of 4 independent biological experiments).

PVR causes a pathologic cell migration into the vitreous and other retinal areas after retinal detachment. To quantify migration, two different experiments were performed. In the scratch wound healing assay, the area repopulated by AM-exposed cells 24 hours after the induced scratch was significantly reduced compared to the control (*p* < 0.001; *n* = 40 technical repeats of 4 independent biological experiments). In addition, migration of AM-exposed cells was attenuated in a Boyden chamber assay (*p* = 0.003; *n* = 28 technical repeats of 4 independent biological experiments) (Figure 2). Thus, both assays are consistent that migration activity is reduced by AM.

### 3.3. Extracellular Matrix Contraction

**Figure 3:**
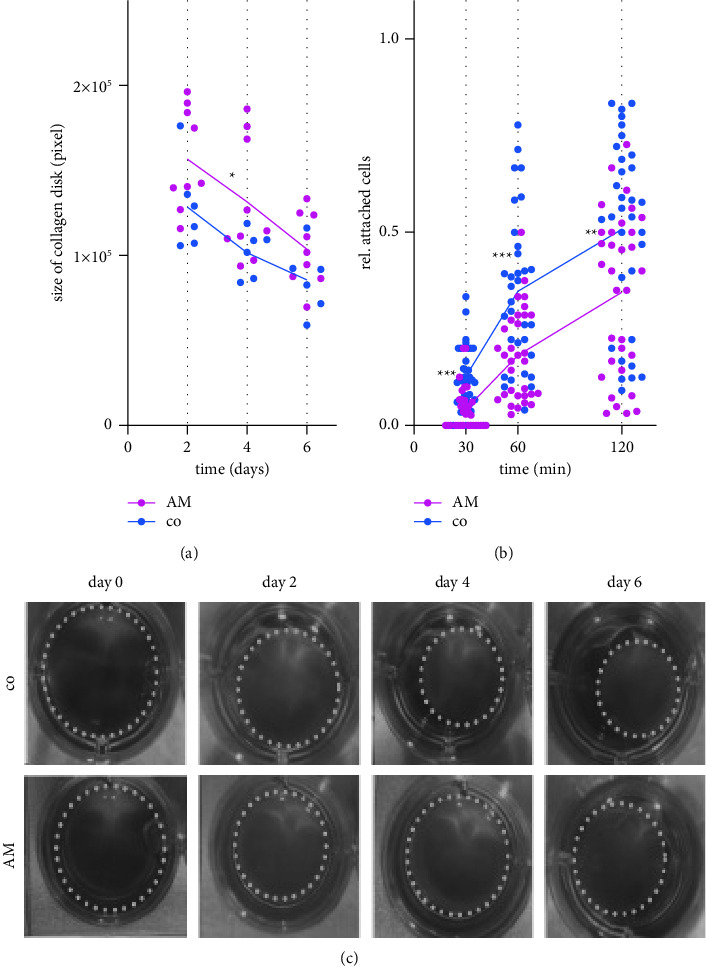
While collagen contraction was largely unaffected, (a) cell adhesion of hPVR was significantly reduced by AM (b), with a statistically significant reduction in adhesion being observed at all time points (*p* < 0.001 at 30 and 60 min; *p*=0.005 at 120 min). At most time points, AM was unable to prevent contraction of collagen discs populated by hPVR cells compared to the untreated control. Shown are representative examples of the collagen slices, including size as measured by circular area projection. (c) ^*∗*^*p*=0.04; ^*∗∗*^*p*=0.005; ^*∗∗∗*^*p* < 0.001; collagen contraction: *n* = 9 technical repeats for AM and *n* = 6 for co of 3 independent biological experiments; cell adhesion: *n* = 32 technical repeats of 4 independent biological experiments.

Through contraction and shortening of retinal and fibrotic tissue, PVR leads to the recurrent retinal detachments. The effect of AM on cell contraction did not play a major role in our experiments. At most time points, AM was unable to affect the contraction of collagen discs populated with hPVR cells compared to the untreated control. A slight statistical significance was found at day 4, when the contraction in the AM group was lower than that in the control group (*p* = 0.04; *n* = 9 technical repeats of 3 independent biological experiments) (Figures 3(a) and 3(c)).

### 3.4. Cell Adhesion

Cell adhesion was examined as a measure of the cells ability to adhere to other tissues after proliferation and migration. The amount of cells adhering to culture plastic after subculture was statistically significantly reduced at all time points when exposed to AM compared to the untreated control (*p* < 0.001 at 30 and 60 min; *p* = 0.005 at 120 min; *n* = 32 technical repeats of 4 independent biological experiments) (Figure 3(b)).

### 3.5. Immunofluorescence Staining

**Figure 4:**
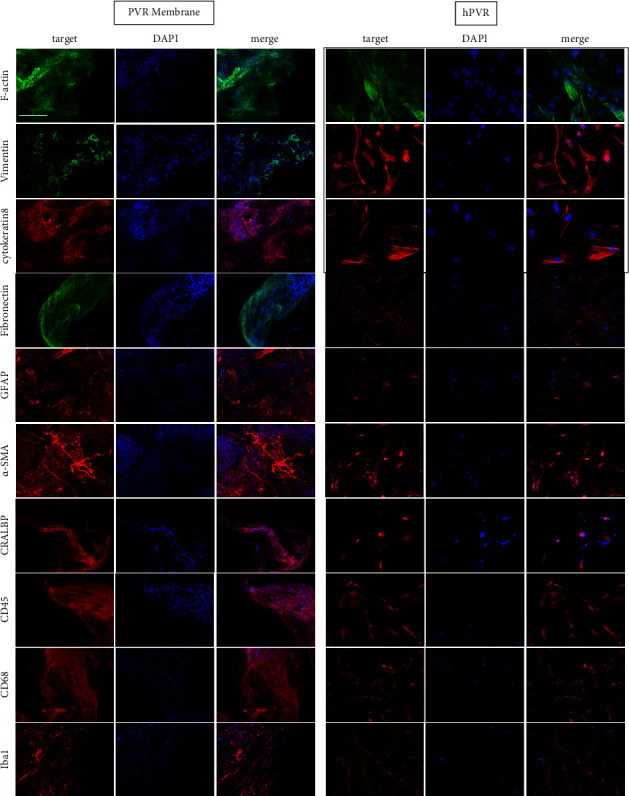
Immunohistochemical characterization of cells on freshly excised PVR membranes and hPVR. PVR membranes and hPVR were stained for RPE, glial, fibroblastic, and immunocellular markers to differentiate the major phenotype in PVR membranes and hPVR (case surrounded images, also seen in Figure 5; *n* = 3 biological experiments; scale bar = 100 *µ*m).

To correlate and characterize cells on PVR membranes and cultured hPVR, immunofluorescence staining for epithelial, glial, fibroblastic, and immunocellular markers was performed. Cells showed staining and morphologic correlation for F-actin, vimentin, fibronectin, and *α*-SMA in PVR membranes similar to hPVR. Primary PVR membrane cells as well as hPVR showed partial staining but no morphologic correlation for the glial specific proteins GFAP and CRALBP. Immune cell involvement was examined by detection of CD45 as a marker for leukocytes and Iba1 and CD68 as markers for microglia and macrophage activation. Protein-specific staining was detected only for Iba1, which was slightly more abundant in PVR membrane cells than in hPVR (*n* = 3 biologicals experiments) (Figure 4).

**Figure 5:**
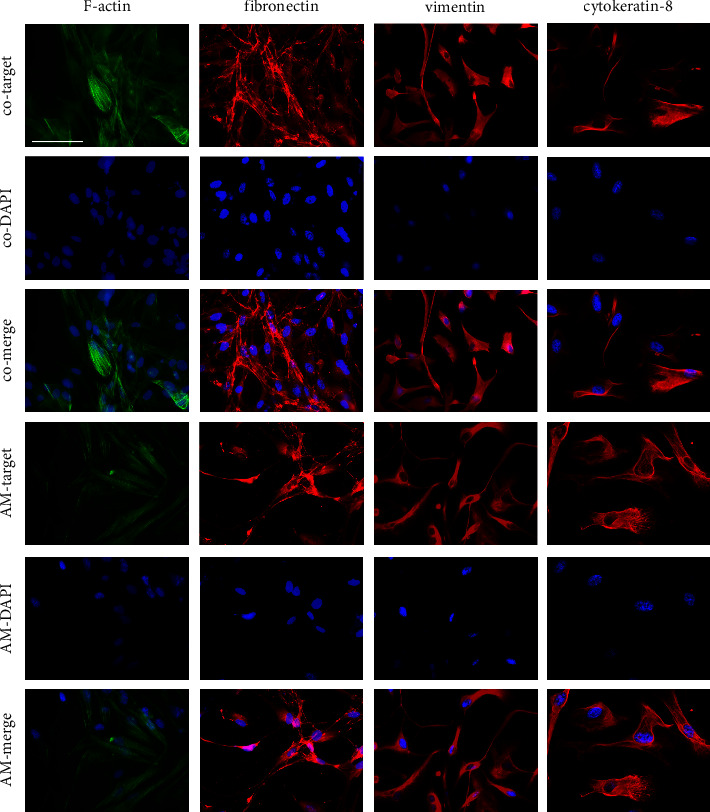
The hPVR cells showed specific immunofluorescence staining for several proteins involved in the process of celltransdifferentiation into myofibroblastic contractile cells. For this purpose, fibronectin, cytokeratin-8, vimentin, and F-actin were stained, and representative images for both the control and AM groups are shown. All stained markers correlated well with the expected fibrotic response in PVR cells, and no induction of fibrosis progression was observed after addition of AM. In contrast, a slight loss of typical cellular pathological PVR properties was observed (*n* = 3 biological experiments; scale bar = 100 *µ*m).

Transdifferentiation of various cell types into myofibroblastic contractile cells has been implicated in the development of PVR. The hPVR cells used in our study showed specific immunofluorescence staining for several proteins involved in this process. Fibronectin appeared as foci located around the plasma membrane. Subjectively, these foci were reduced after exposure to AM. Cytokeratin-8, vimentin, and F-actin are proteins of the cytoskeleton. All were detected as intracytoplasmic filaments, suggesting a myofibroblast-like phenotype possibly originating from the retinal pigment epithelium. Vimentin and F-actin were subjectively reduced after AM exposure compared with the controls. Cytokeratin-8, a marker of an epithelial cell phenotype, was equally abundant in controls and AM. All stained markers correlated well with the expected fibrotic response in PVR cells. AM did not lead to fibrosis progression, but rather a slight loss of typical cellular pathological PVR characteristics was observed (*n* = 3 biologicals experiments) (Figure 5).

### 3.6. Western Blot

**Figure 6:**
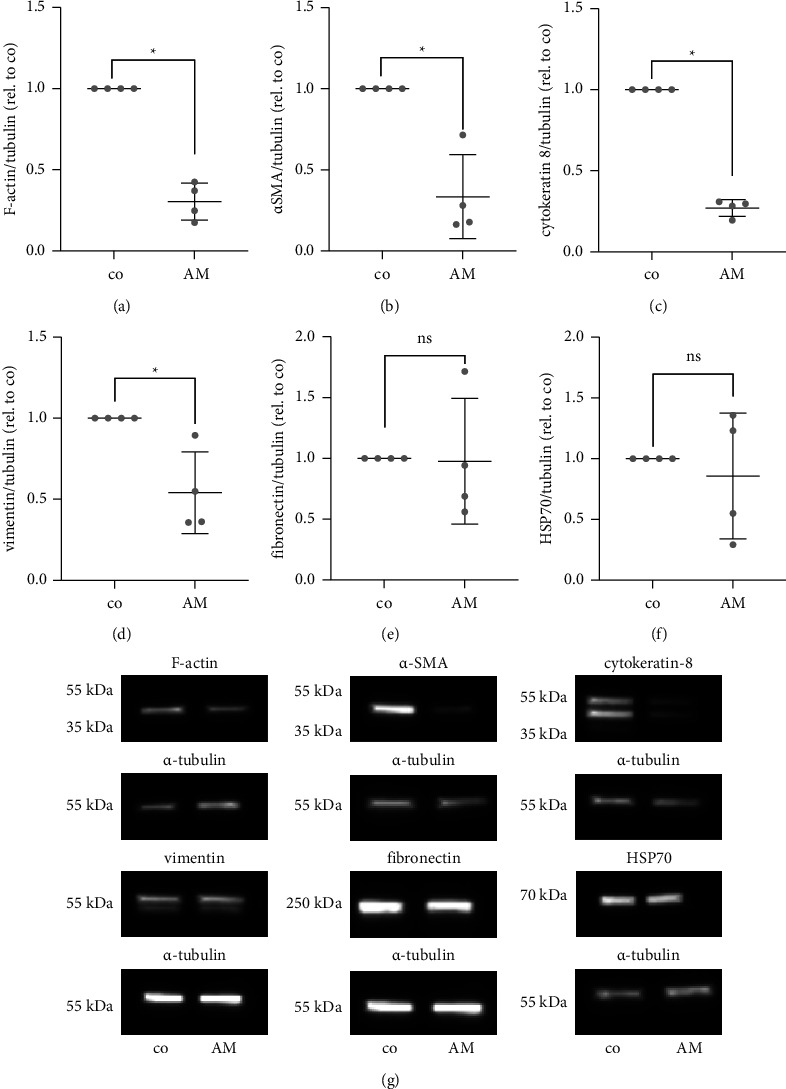
In the Western blot, AM caused a reduction in protein expression of myofibroblastic proteins in hPVR cells. (a–f) When compared to the control, F-actin was significantly reduced 3.3-fold (*p* = 0.03), *α*-SMA was significantly reduced 3-fold (*p* = 0.03), cytokeratin-8 was significantly reduced 3.7-fold (*p* = 0.03), and vimentin was significantly reduced 1.8-fold (*p* = 0.03). Fibronectin (*p* = 0.3), and HSP-70 (*p* > 0.99) did not reach a statistical significant change. (g) Representative presentation of protein expression change following AM incubation (*n* = 4 biological independent experiments).

In PVR, cells undergo myofibroblastic transdifferentiation. To determine whether AM has an effect on the myofibroblastic phenotype and to verify the observed changes in immunofluorescence staining, typical protein levels expressed by the hPVR were evaluated by a Western blot assay. Compared to the control, F-actin was significantly reduced by 3.3-fold (*p* = 0.03), *α*-SMA by 3-fold (*p* = 0.03), cytokeratin-8 by 3.7-fold (*p* = 0.03), and vimentin by 1.8-fold (*p* = 0.03) in AM-exposed cells. No statistically significant changes were observed for fibronectin (*p* = 0.3) and HSP70 (*p* > 0.99). These data suggest that hPVR cells express less myofibroblastic markers when exposed to AM (*n* = 4 biological independent experiments) (Figure 6 and Supplementary Figure 1).

### 3.7. Proteomics

**Figure 7:**
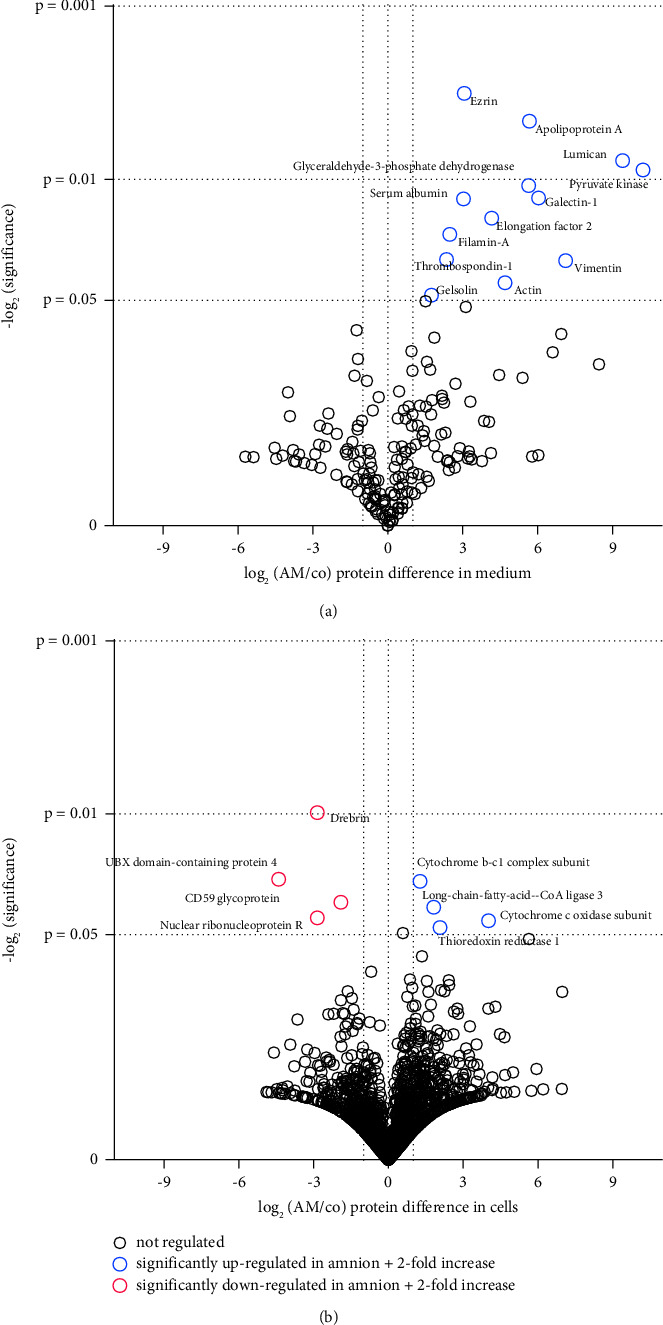
Volcano plot of the results of the proteome analysis. Several proteins are significantly regulated throughout the whole proteome analysis. (a) Secreted proteins in the cell culture medium and (b) proteins inside the cells have been investigated. A *p* value <0.05 and at least 2-fold difference between the two study groups were considered significant (*n* = 4 biological independent experiments).

To investigate the effect of AM on the hPVR cells, a detailed analysis of phenotypic changes and proteome expression was performed. This was achieved by a large-scale proteomic analysis in which changes in protein levels were determined both in the culture medium and in the cells. An untreated control was compared with AM-exposed hPVR cells. Protein expression analysis by label-free/liquid chromatography-tandem mass spectrometry identified 2,657 proteins. Based on more than 2-fold increase in expression and statistical significance, 21 proteins were upregulated in AM-treated cells and 17 were observed exclusively in this group (Figure 7). The significantly regulated proteins are mainly related to cytoskeleton, lipid metabolism, cell-matrix contraction, and protein folding (*n* = 4 biological independent experiments) (Table 1).

### 3.8. Cell Viability

To exclude the possibility that the results of this study were caused by toxic effects of AM on hPVR, three assays were performed. In the WST assay, the viability of AM-exposed cells was not significantly reduced compared to the control (*p* = 0.2; *n* = 9 technical repeats of 3 independent biological experiments). There was also no increase in dead cells in a dye exclusion assay. As a positive control, methanol incubation significantly increased the number of dead cells (*n* = 3 technical repeats of 3 independent biological experiments).

**Figure 8:**
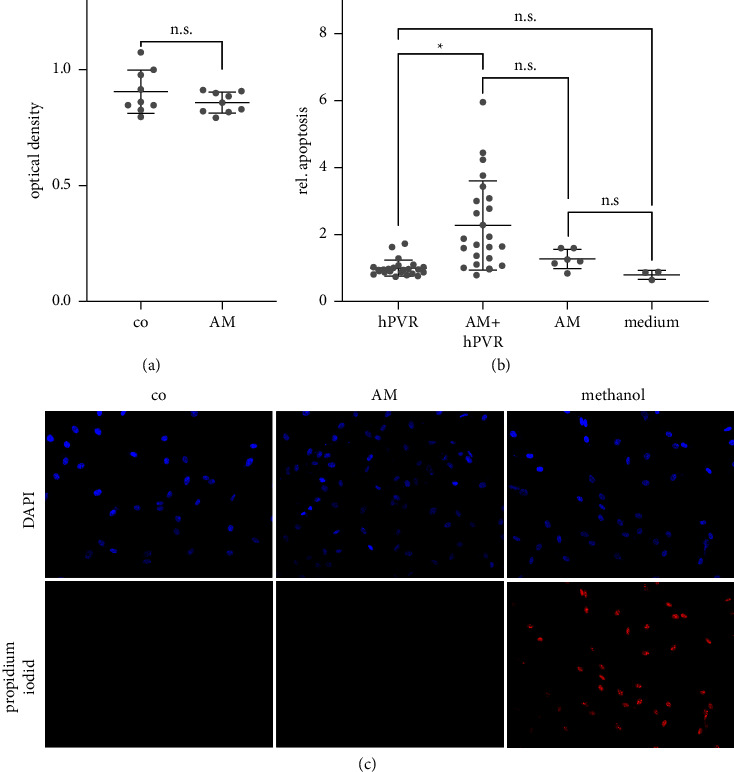
Toxicity as a reason for the effects observed in the other experiments was excluded in this study. In the WST viability test (a), no difference was observed between AM and the control (*p* = 0.2). In addition, there were no dead cells in the dye exclusion test in both AM and control (c). Histone-complexed DNA fragments (b) were significantly increased in AM-treated cells compared to control (*p* = 0.01). In this experiment, we could not determine whether this increase was due to the cells in AM that may have undergone apoptosis. (^*∗*^*p*=0.01; WST assay: *n* = 9 technical repeats of 3 independent biological experiments; dye exclusion assay: *n* = 3 technical repeats of 3 independent biological experiments; Histon complexed DNA ELISA: *n* = 24 technical repeats for hPVR and AM + hPVR, *n* = 6 technical repeats for AM only, and *n* = 3 for medium only, all of 3 independent biological experiments).

In contrast, however, histone-complexed DNA fragments were significantly higher in AM-treated cells (*p* = 0.01). In this experiment, we were unable to determine whether this increase was due to the AM possibly undergoing apoptosis during preparation and freezing, as apoptosis was not increased in AM alone compared to medium alone (*p* = 0.7). There was also no significant increase in apoptosis between medium alone and hPVR alone (*p* = 0.2) and no significant increase between AM and AM plus hPVR cells (*p* = 0.2; *n* = 24 technical repeats for hPVR and AM + hPVR; *n* = 6 technical repeats for AM only; and *n* = 3 for medium only, all of 3 independent biological experiments) (Figure 8).

## 4. Discussion

In PVR, fibrotic contractile cells invade at various sites, migrate, proliferate, and produce extracellular matrix to form membranes that can shorten and detach the retina [27]. Several cell phenotypes have been identified as precursors of these transdifferentiated myofibroblasts. These include, for example, retinal pigment epithelial cells, glial cells, and immune cells. They are activated and modulated by various growth factors and cytokines [28]. Although AM has been shown to be a highly versatile surgical tool, its mode of action on the proposed pathologic basis of PVR in potential intravitreal implantation has been unclear, as it may promote either an activating, suppressive, or no effect on PVR progression. In the primary patient-derived PVR myofibroblasts (hPVR) used for this study, we demonstrated an inhibitory and regenerative effect of AM on PVR. After incubation with AM, hPVR showed significantly reduced fibrotic properties as determined by migration, proliferation, and cell adhesion, which may be attributed to the stabilizing effect of AM. Immunofluorescence showed a reduced expression of the fibrosis markers, which was confirmed by our Western blot. Proteomics also revealed significant changes in potentially fibrosis-inducing mediators in hPVR, mainly related to the cytoskeleton, lipid metabolism, extracellular matrix contraction, and protein folding. This *in vitro* work suggests PVR-inhibiting properties of AM or at least negates the induction of fibrosis progression that would make its use contraindicated in other vitreoretinal diseases.

Previous clinical findings from several case series are consistent with our laboratory results. Treatment of persistent macular hole by an AM patch transplanted during pars plana vitrectomy is well known [29]. However, few clinical case reports have been published on the use of AM for retinal detachment. The use of intraocular AM in complicated retinal detachment cases has been reported in small numbers by various groups [7, 30]. It seems that AM was well tolerated and the cases demonstrated an acceptable clinical course [7, 30–32].

Although early clinical data show theoretical benefits and our results suggest no contraindication for a clinical application of AM in retinal diseases, the intraocular use of AM is still far from clinical routine. For PVR, pars plana vitrectomy with careful membrane removal, retinal reattachment, and vitreous tamponade will continue to be the treatment of choice [3]. However, epiretinal AM may be a tool to improve the rate of postoperative PVR in selected cases. The mode of application is still unclear; whole membrane implantation [7–9] or AM secretomes [33] have been described. In the case of secretomes, application in the form of a single or repeated intravitreal injection would be conceivable. Mechanical properties of epiretinal AM may also play a role in the PVR suppression [34, 35]. Therefore, we would most likely opt for this option.

As from our perspective, it is not yet clear whether explantation of M is necessary, and it is unclear at what time explantation should be initiated. After the release of the biologically active factors, AM is probably no longer useful or could be visually disturbing and have adverse effects if it floats in the eye. A possible suggestion would be to remove the membrane at the time of a possible silicone-oil tamponade removal, which is often used in these severe PVR cases that could also benefit from AM transplantation.

In line with our results, the main effects of this application were further investigated *in vitro*. RPE cells undergo epithelial-mesenchymal transformation as part of PVR, which could be prevented by the phenotype-stabilizing effect of AM [36]. In addition, RPE cells have been shown to form dense colonies on AM that maintain their epithelial phenotype [37, 38]. *In vivo* studies in mice and rats [39] suggest a neuroprotective effect against damage to the peripheral and central nervous system, which would include the retina. In rats suffering from uveitis, subretinal AM transplantation attenuated the inflammatory response and contributed to the preservation of retinal structure [40].

Immunofluorescence, proteomics, and Western blot analysis suggest that the main findings observed may be due to extensive changes in the cytoskeleton. The cytoskeleton is a dynamic network that is constantly remodeled by many different stimuli and is involved in a variety of cellular functions. Alterations in the cytoskeleton have been implicated in the development of PVR. For example, a complete proteomic analysis of vitreous samples obtained during pars plana vitrectomy in PVR eyes revealed alterations of several cytoskeleton-associated proteins [41]. Epithelial-mesenchymal transformation has been identified as a hallmark of PVR [42], and alterations in the actin cytoskeleton, particularly upregulation of the protein *α*-SMA, have been widely used as markers of epithelial-mesenchymal transformation [43].

Consistent with other findings in the literature and as seen in the proteomic analysis, in addition to cytoskeleton-related proteins, proteins involved in lipid metabolism and other metabolic pathways were also affected by AM. Simvastatin, a 3-hydroxy-3-methylglutaryl coenzyme A reductase inhibitor, belongs to the statin group and is commonly used to treat hypercholesterolemia. It reduced PVR formation in the rabbit model [44] and in human RPE cells [45, 46]. Clinical evidence of a beneficial effect against PVR after retinal detachment has also been found. Concentrations of several growth factors in the vitreous of statin-treated retinal detachment patients were lower after vitrectomy, and an improvement in visual acuity was observed [47]. In a Finnish population-based cohort study of vitrectomized patients with rhegmatogenous retinal detachment, the risk of repeat vitrectomy was 28% lower when systemic statins were used [48]. The clinical findings and observations of protein regulation in lipid metabolism and other metabolic pathways associated with a PVR reduction should be investigated in further studies.

It should be noted that the experimental data of this study are limited to the nature of an *in vitro* setting. The hPVR cells used are a very simplified model of PVR and do not fully represent the pathologic basis of PVR, as the retina is a complex tissue with glial cells, neuronal tissue, immune responses, and blood vessels. The cell culture environment also does not fully represent the conditions in the eye after retinal detachment and breakdown of the aqueous humor barrier. Therefore, the hPVR model does not guarantee complete translation to the clinical situation. Other limitations include the lack of ocular pharmacokinetics, which may result in lower or higher concentrations of cytokines, growth factors, and extracellular matrix components. All of these limitations may have led to an overestimation or underestimation of the observed effects compared to the clinical situation, and future studies need to verify our results.

In conclusion, this *in vitro* work suggests possible PVR-inhibitory properties of AM, while it does not show induction of fibrosis progression that would make its use contraindicated in the applications discussed. Further studies are needed to assess the clinical therapeutic relevance.

## Figures and Tables

**Table 1:** Classification of *x*-fold regulation and significances of regulated proteins.

Significantly regulated protein	*p* value	*x*-fold	Related to
*Upregulated medium (AM/co)*			
Serum albumin	0.01	8.2	Carrier protein
Gelsolin	0.046	3.4	Cytoskeleton
Ezrin	0.003	8.4	Cytoskeleton
Apolipoprotein A	0.005	51	Lipid metabolism
Glyceraldehyde-3-phosphate dehydrogenase	0.008	1197	Lipid metabolism
Thrombospondin-1	0.03	5.1	Cell-matrix interaction
Vimentin	0.03	139	Cytoskeleton
Galectin-1	0.01	66	Cell-matrix interaction
Elongation factor 2	0.02	18	Gene expression
Pyruvate kinase	0.01	50	Carbohydrate metabolism
Filamin-A	0.02	5.6	Cytoskeleton
Lumican	0.008	678	Cell-matrix interaction
Actin	0.04	26	Cytoskeleton

*Exclusive proteins to AM in the medium*			
Alpha-actinin-4	0.009		Cytoskeleton
Calumenin	0.02		Protein folding
Filamin-B	0.003		Cytoskeleton
Phosphoglycerate kinase 1	0.03		Carbohydrate metabolism
Alpha-1-antitrypsin	0.01		Protein folding
Serotransferrin	0.02		Iron transport
Profilin-1	0.047		Cytoskeleton
Heat shock protein HSP 90-alpha	0.04		Protein folding
Heat shock cognate 71 kDa protein	0.047		Protein folding
Alpha-actinin-1	0.02		Cytoskeleton
Calpain-2 catalytic subunit	0.005		Cytoskeleton
Protein disulfide-isomerase A3	0.02		Protein folding
Triosephosphate isomerase	0.02		Carbohydrate metabolism
14-3-3 protein epsilon	0.03		Signaling
Tropomyosin alpha-4 chain	0.04		Cytoskeleton
Tubulin alpha-1B chain	0.01		Cytoskeleton
Tubulin beta-4B chain	0.046		Cytoskeleton
Peroxiredoxin-1	0.047		Redox regulation

*Upregulated in cells (AM/co)*			
Thioredoxin reductase 1	0.045	4.2	Redox regulation
Long-chain-fatty-acid-CoA ligase 3	0.03	3.5	Lipid metabolism
Cytochrome c oxidase subunit	0.04	16	Respiratory chain
Cytochrome b-c1 complex subunit	0.02	2.4	Respiratory chain

*Downregulated in cells (AM/co)*			
CD59 glycoprotein	0.03	3.7	Cell survival
Nuclear ribonucleoprotein R	0.04	7.2	Gene expression
Drebrin	0.009	7.2	Cytoskeleton
UBX domain-containing protein 4	0.045	21	Protein folding

Each protein was also categorized according to its function. The following functional categories were regulated according to the number of proteins involved: cytoskeleton: 14, protein folding: 6, lipid metabolism: 3, cell-matrix interaction: 3, carbohydrate metabolism: 2, redox regulation: 2, respiratory chain: 2, gene expression: 2, others: 5. Thus, proteins related to cytoskeleton, lipid metabolism, cell-matrix contraction, and protein folding were most significantly regulated.

## Data Availability

Data are included within the article.

## Supplementary Materials

Supplementary Materials Supplementary Figure 1: demonstration of *n* = 4 biological repeats of the Western blot analysis. AM caused a reduction in protein expression of myofibroblastic proteins in hPVR cells.
