# Supplementary figures and images for: Epiretinal Amniotic Membrane Influences the Cellular Behavior of Profibrotic Dedifferentiated Cells of Proliferative Vitreoretinopathy In Vitro
Source: J Tissue Eng Regen Med. 2023 Oct 18;2023:8820844. doi: 10.1155/2023/8820844 (PMC11918902; doi:10.1155/2023/8820844)

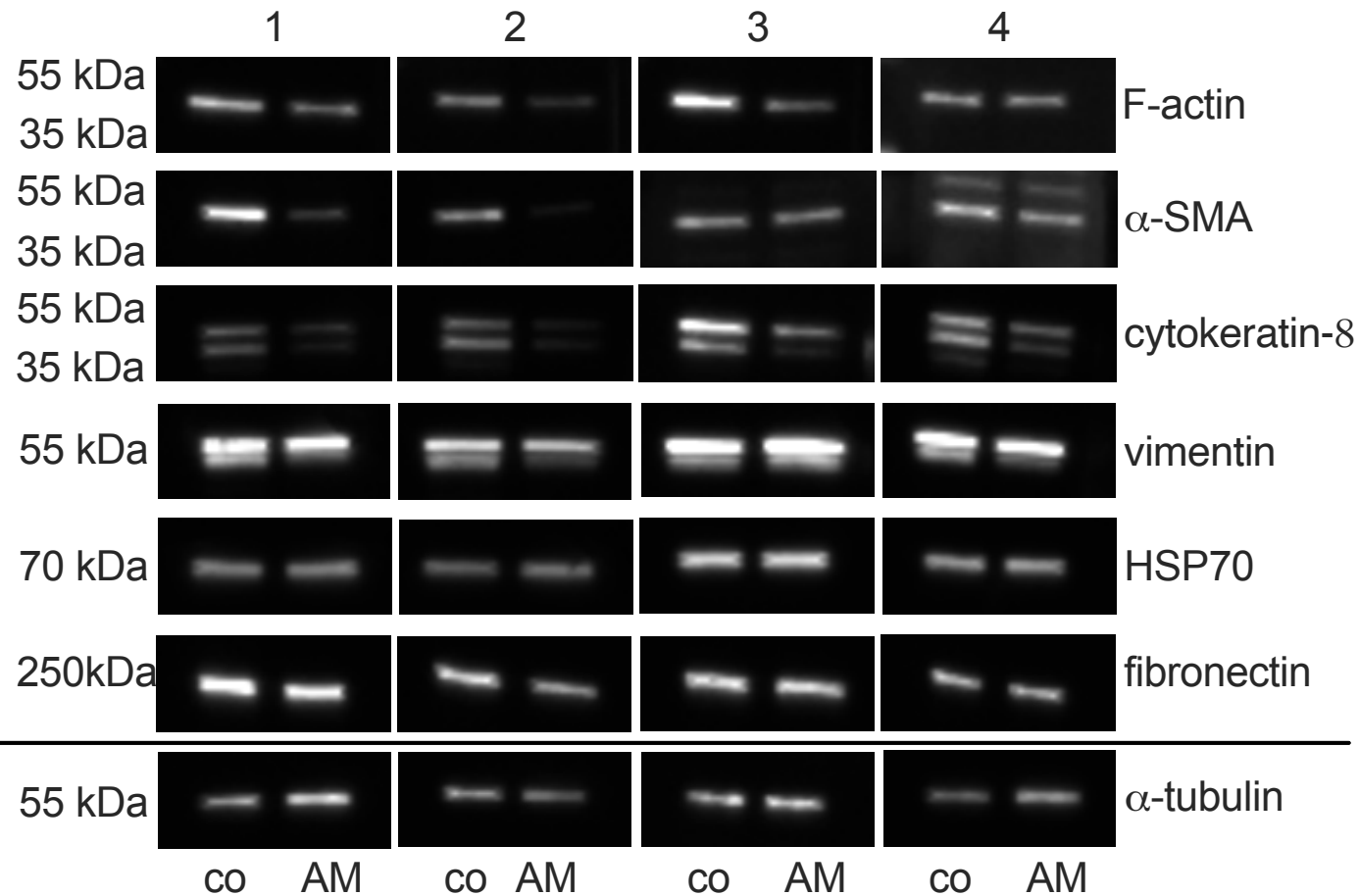

Supplement: Supplementary Materials — Supplementary Figure 1: demonstration of n = 4 biological repeats of the Western blot analysis. AM caused a reduction in protein expression of myofibroblastic proteins in hPVR cells. [file 8820844.f1.pdf]
